# Metabolism of amino acids differs in the brains of Djungarian hamster (*P. sungorus*) and Roborovskii hamster (*P. roborovskii*)

**DOI:** 10.1186/2193-1801-3-277

**Published:** 2014-06-02

**Authors:** Hiromi Ikeda, Takahiro Kawase, Mao Nagasawa, Vishwajit Sur Chowdhury, Shinobu Yasuo, Mitsuhiro Furuse

**Affiliations:** Laboratory of Regulation in Metabolism and Behavior, Faculty of Agriculture, Kyushu University, Fukuoka, 812-8581 Japan; Division for Arts and Science, Faculty of Arts and Science, Kyushu University, Fukuoka, 819-0395 Japan

**Keywords:** L-Tyrosine, D-Tyrosine, Djungarian hamsters, Roborovskii hamsters, Brain

## Abstract

Djungarian hamster (*P. sungorus*) and Roborovskii hamster (*P. roborovskii*) belong to the same genus of *phodopus*. Roborovskii hamster shows high locomotor activity and low level of dopamine (DA) in the brain. Administration of L-tyrosine, a precursor of DA, decreases locomotor activity in Roborovskii hamsters. However, the amino acid metabolism in relation to the hyperactivity is not yet well known. In the present study, L- and D-amino acid concentrations in the brain, liver, and plasma in Djungarian and Roborovskii hamsters were investigated during day and night times to explain the possible difference in hyperactivity between them. Most of the examined amino acids were higher in the night time when hamsters are active compared to those in day time. L- and D-tyrosine concentrations were higher in the liver of Roborovskii hamsters than in Djungarian hamsters. Furthermore, brain concentration of D-tyrosine was higher in the Roborovskii than in Djungarian hamsters, but no significant difference was observed for L-tyrosine concentrations between the two species. These results suggest that the conversion of L-tyrosine to D-tyrosine in the brain of Roborovskii hamster may be higher than in Djungarian hamster, which may cause low DA concentration and hyperactivity in Roborovskii hamster. On the other hand, L- and D-serine, which are known as sedative factors, were lower in Roborovskii hamsters than Djungarian hamster. These results suggest that species-specific regulation in amino acid metabolism may contribute to hyperactivity in Roborovskii hamsters.

## Introduction

Hyperactivity is one of the symptoms of some dysfunctions like attention-deficit hyperactivity disorder (ADHD) in humans or problematic behaviors of companion animals. ADHD is characterized by hyperactivity together with inattention and impulsivity (Spencer et al. 2007). Approximately 8 to 12% children are suffering from this disorder worldwide (Biederman and Faraone 2005). Because some people cannot recognize ADHD as a mental disorder, the total number of ADHD patient may be underestimated. It is considered that dopamine (DA)-based psycho-stimulants may be involved for the occurrence of ADHD, but this mechanism is still unknown (Leo et al. 2003).

Djungarian hamster (*Phodopus sungorus*) and Roborovskii hamster (*Phodopus roborovskii*) belong to the same genus of *Phodopus*. Although the sexual maturation and the duration of the pregnancy are similar in these two species, their behaviors show differences (Miller 1910; Ross 1994, 1998; Kabuki et al. 2008). Djungarian hamsters are tame, but Roborovskii hamsters show hyperactivity. Thus Djungarian hamster, but not Roborovskii hamster, is generally used as a laboratory animal, although both hamsters are popular as a companion animal. Hamsters have been used as animal model for human clinical studies (Jennings et al. 1976). Roborovskii hamster has low brain dopamine level which is comparable with ADHD patients who also show low dopamine level in the brain (Viggiano et al. 2004). Phodopus species have been used as important laboratory organisms. They provide the opportunity to study as an excellent model system for the evolution of hormonal control of photoperiod and seasonal physiology as well as behaviors (Feoktistova et al. 2010). However, the mechanism of tameness or hyperactivity between these two species has not yet been fully understood. Roborovskii hamsters can be suggested as a model animal of ADHD, and the comparison between these two hamsters may help to understand the underlying mechanism of behavioral differences.

Now-a-days a lot of people have companion animals, and sometimes they face problems due to their abnormal behaviors including stereotypical behavior induced by stress. Our previous study demonstrated that hyperactivity in Roborovskii hamsters associated with low DA concentration in its brain compared to Djungarian hamsters (Kabuki et al*.*^2008^). When L-3,4-dihydroxyphenylalanine (L-DOPA), a precursor of DA, was administered to Roborovskii hamsters, DA level increased in the brain and locomotor activity conversely decreased in a dose-dependent manner (Kabuki et al*.*2009a). In case of a single administration of L-tyrosine, a precursor of L-DOPA, modified brain monoamine metabolism increased DA turnover rate, but did not ameliorate signs of hyperactivity in the Roborovskii hamsters (Kabuki et al*.*2009b). On the other hand, chronic administration of L-tyrosine significantly decreased locomotor activity in the Roborovskii hamsters, when they were reared in home cage, with higher metabolic turnover rate of DA and norepinephrine (NE) (Kabuki et al*.*^2011^). These findings suggest that L-tyrosine contents and its metabolism in the brain may have some important roles for the induction of hyperactivity.

Most of the amino acids can occur in two isomeric forms. By convention, these are called L- and D-isomeric forms, analogous to left-handed and right-handed configurations (Hoon et al. 1986). In the amino acid metabolism, L-amino acids can be racemized or converted to their mirror image configuration, the D-isomers (Friedman et al. 1981). In the present study, we therefore analyzed the contents of L-tyrosine and its optical isomer D-tyrosine in the brain, liver and plasma in Roborovskii and Djungarian hamsters to compare the ratio of these two isomers in connection with their activity behavior between them. Other amino acids contents were also determined to clarify the differences in amino acid metabolism between the two hamsters.

## Material and methods

### Animals and housing conditions

Male Djungarian hamsters (n = 14) and Roborovskii hamsters (n = 14), 3 weeks of age, were purchased from a local dealer similarly as reported previously (Kabuki et al. 2008, 2009a,[b], 2011). We used the minimum number of animals in each group considering the statistical power of sample size and reared under controlled environments. They were housed in a group of three or four animals per cage and freely given access to a standard diet (MF; Oriental Yeast, Tokyo, Japan) and water. A 12-h day/night cycle was maintained throughout the experiment, with light on at 08:00 and off at 20:00. Room temperature was maintained at 23 ± 1°C and humidity at 60%. This study was conducted according to the guidelines for animal experiments in the Faculty of Agriculture and on the Graduate Course of Kyushu University, and to Law No. 105 and Notification No. 6 of the government.

### Tissue preparation

After an acclimation period of 8 days, the brains of Djungarian hamsters and Roborovskii hamsters were obtained during the day time (14:00) when hamsters are resting and the night time (02:00) when hamsters are active (n = 7 in each group). Hamsters were decapitated under anesthesia with isoflurane (Escain®, Mylan, Osaka, Japan), and trunk blood was collected each time. The whole brains were carefully taken out from the skulls and placed on a cold glass dish. The livers were taken, and washed with saline. The plasma separated from brood, which was centrifuged at 4°C, 3000 × g for 20 min. The tissue samples were frozen in liquid nitrogen, and all the samples were stored at −80°C until analysis.

### Amino acid analysis

Amino acid contents in the brains, livers and plasma were analyzed according to the previously described method (Ohmori et al. 2011). The tissues were weighed and homogenized in a solution of 0.2 M ice-cold perchloric acid containing 0.01 mM EDTA 2Na. Samples were allowed to sit on ice for 30 min for deproteinization. The homogenate was centrifuged at 20,000 × g for 15 min. Supernatants were adjusted to pH 3 with 1 M sodium acetate and were filtered through a 0.2-μm filter (Millipore, Bedford, MA, USA). Plasma was prepared by centrifuging it at 20,000 × g for 15 min at 4°C (MX-307, Tommy, Japan), and then it was filtrated through ultrafiltration tubes (Millipore, Bedford, USA). Each 20-μl sample of the brain or liver was dissolved with 2 μl of 1 M NaOH and then vortexed. Both the L- and the D-amino acid contents were measured by a UPLC (the Acquity™ UPLC system comprised of Waters Binary Solvent Manager, Water Sample Manager and Waters FLR Detector) with an ACCQ-TAG™ ULTRA C18 1.7 μm 2.1 × 100 mm column (Waters Corporation, USA). The excitation and emission wavelengths for fluorescent detection of amino acids were 350 nm and 450 nm, respectively. The system was operated with a flow rate of 0.25 ml/min at 30°C. The UPLC gradient system (A = 50 mM sodium acetate (pH 5.9), B = methanol) was 10–20% B over 3.2 min, 20% B for 1 min, 20–40% B over 3.6 min, 40% B for 1.2 min, 40–60% B over 3.8 min, 60% B for 1 min, and 60–10% B over 0.01 min. Just before the analysis in UPLC, each sample (10 μl) was transferred to a UPLC tube, and NAC/OPA (20 μl) and a borate buffer (70 μl) were added; then it was left for 2 min in a dark room. The same method was used for the standard solutions containing 16 L-amino acids, 16 D-amino acids, glycine, taurine and so on. The plasma amino acid concentrations were expressed in nmol/ml, and the amino acid concentrations in the brains were expressed as pmol/mg wet tissue.

### Statistical analysis

Data were analyzed using two-way ANOVA followed by Fishers’ PLSD. All data in each group were first subjected to a Thompson rejection test to eliminate outliers (*P* < 0.01), and the remaining data were used for the analysis among groups.

## Results

**Figure 1:**
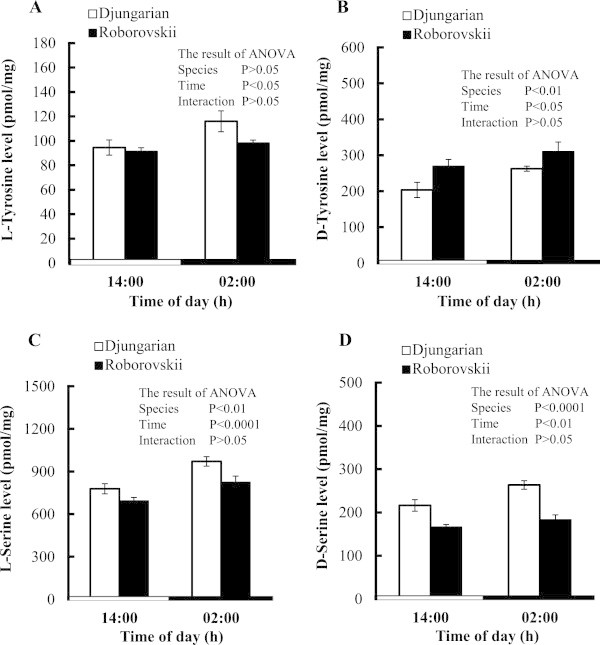
**Changes in the concentrations of brain L-tyrosine (A), D-tyrosine (B), L-serine (C) and D-serine (D) in Djungarian and Roborovskii hamsters.** Data are expressed as means ± S.E.M. n = 6–7/group.

In the brain, both L- and D-tyrosine as well as L- and D-serine levels were significantly (*P* < 0.05) higher at night than day time. D-Tyrosine level was significantly (*P* < 0.01) higher in Roborovskii than in Djungarian hamsters (Figure 1B), but no significant difference was found in L-tyrosine between the two species (Figure 1A). Both L- and D-serine levels were significantly (*P* < 0.01) higher in Djungarian than Roborovskii hamsters (Figure 1C,D).

**Figure 2:**
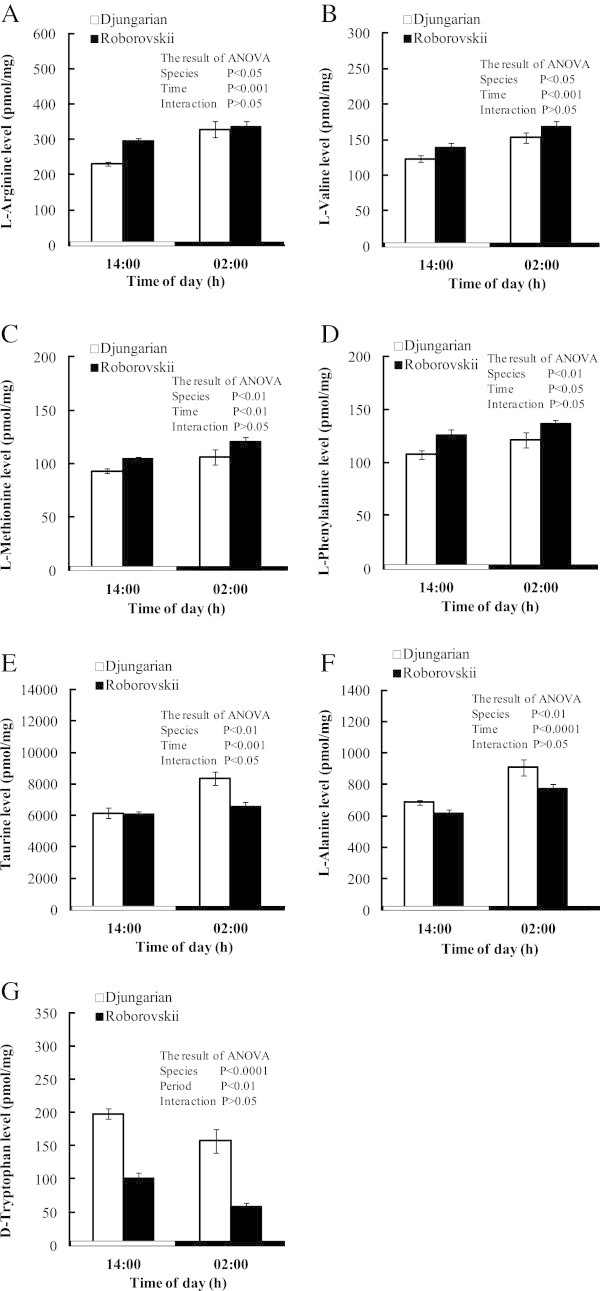
**Changes in the concentrations of several brain L- and D-amino acids (L-arginine (A), L-valine (B), L-methionine (C), L-phenylalanine (D), taurine (E), L-alanine (F) and D-tryptophan (G)) in Djungarian and Roborovskii hamsters.** Data are expressed as means ± S.E.M. n = 5–7/group.

Figure 2 shows the changes of the several free amino acids in the whole brain between the two species. Most of the amino acids were significantly (*P* < 0.05) higher in the night time than in the day time. However, only D-tryptophan decreased in both hamsters during the night time (Figure 2G). L-Arginine, L-valine, L-methionine and L-phenylalanine levels were significantly higher in Roborovskii than Djungarian hamsters (Figure 2A,B, C and D), but the reverse was observed for taurine, L-alanine and D-tryptophan (Figure 2E,F and G). Table 1 shows the concentrations of the free amino acids in the brain of two hamsters which were changed only by the effect of time and/or interaction without L-tryptophan. Significant interactions between the species and time were detected in L-glutamine, L-histidine and GABA, implying that the values of these three amino acids increased during the night time in both hamsters.

**Table 1:** **Concentrations of L- and D-amino acids in the brain of Djungarian and Roborovskii hamsters during light and dark periods**

Amino acids	Djungarian hamster		Roborovskii hamster		Results of ANOVA		
	Light	Dark	Light	Dark	Species	Time	Interaction
*Essential amino acids*							
L-Isoleucine	63.5 ± 2.3	74.7 ± 3.9	69.2 ± 2.8	80.1 ± 3.8	NS	*P* < 0.01	NS
L-Leucine	172 ± 7	220 ± 12	202 ± 6	234 ± 14	NS	*P* < 0.001	NS
L-Histidine	636 ± 28	815 ± 44	697 ± 28	732 ± 23	NS	*P* < 0.01	*P* < 0.05
L-Tryptophan	2323 ± 149	2677 ± 176	2488 ± 156	2758 ± 324	NS	NS	NS
*Nonessential amino acids*							
GABA	2708 ± 102	3881 ± 185	3107 ± 88	3634 ± 122	NS	*P* < 0.0001	*P* < 0.05
L-Aspartic acid	2463 ± 63	3045 ± 97	2637 ± 106	3183 ± 158	NS	*P* < 0.001	NS
D-Aspartic acid	13.9 ± 1.2	18.0 ± 0.8	14.6 ± 0.6	17.3 ± 0.5	NS	*P* < 0.05	NS
L-Glutamine	6882 ± 176	9264 ± 691	7248 ± 264	7696 ± 317	NS	*P* < 0.01	*P* < 0.05

Values are means ± S.E.M. in pmol/mg wet tissue; *n* = 5–7 in each group; NS: Not significant.

Table 2 shows all the free amino acid concentrations analyzed in the liver of Roborovskii and Djungarian hamsters during day and night times. Both L-tyrosine and D-tyrosine concentrations were higher in Roborovskii than in Djungarian hamsters. Similar pattern of changing was observed in the other free amino acid concentrations in the liver of Roborovskii and Djungarian hamsters. Free amino acid concentrations were significantly higher in the liver at night than the day time except for D-aspartic acid, L-glutamine, L-histidine and GABA. Significant interactions between species and times were detected in taurine, L-alanine, L-tyrosine, L-valine and L-isoleucine, suggesting that the darkness stimulates more for greater free amino acid concentrations in Roborovskii hamsters.

**Table 2:** **Concentrations of L- and D-amino acids in the liver of Djungarian and Roborovskii hamsters during light and dark periods**

Amino acids	Djungarian hamster		Roborovskii hamster		Results of ANOVA		
	Light	Dark	Light	Dark	Species	Time	Interaction
*Essential amino acids*							
L-Valine	375 ± 24	518 ± 40	510 ± 22	867 ± 69	*P* < 0.01	*P* < 0.01	*P* < 0.05
L-Histidine	1978 ± 346	1672 ± 190	2530 ± 151	2912 ± 238	*P* < 0.01	NS	NS
L-Methionine	99 ± 7	162 ± 16	181 ± 9	333 ± 38	*P* < 0.01	*P* < 0.01	NS
L-Tryptophan	207 ± 19	242 ± 6	260 ± 11	334 ± 24	*P* < 0.01	*P* < 0.01	NS
L-Phenylalanine	183 ± 5	266 ± 13	318 ± 16	483 ± 44	*P* < 0.01	*P* < 0.01	NS
L-Leucine	548 ± 31	783 ± 53	747 ± 33	1218 ± 100	*P* < 0.01	*P* < 0.01	NS
L-Isoleucine	203 ± 14	273 ± 19	274 ± 11	453 ± 36	*P* < 0.01	*P* < 0.01	*P* < 0.05
*Nonessential amino acids*							
L-Serine	516 ± 45	798 ± 81	682 ± 19	1212 ± 114	*P* < 0.01	*P* < 0.01	NS
Taurine	1925 ± 180	2116 ± 181	2282 ± 125	3474 ± 340	*P* < 0.01	*P* < 0.01	*P* < 0.05
L-Aspartic acid	315 ± 22	384 ± 34	732 ± 30	840 ± 44	*P* < 0.01	*P* < 0.05	NS
D-Aspartic acid	17.3 ± 0.9	16.8 ± 1.0	26.8 ± 1.7	27.5 ± 1.1	*P* < 0.01	NS	NS
L-Alanine	4139 ± 564	4064 ± 288	5220 ± 136	7112 ± 569	*P* < 0.01	*P* < 0.05	*P* < 0.05
L-Glutamine	4598 ± 269	5142 ± 603	6837 ± 478	6328 ± 222	*P* < 0.01	NS	NS
L-Arginine	18.9 ± 2.0	27.9 ± 2.6	41.4 ± 1.9	45.5 ± 3.3	*P* < 0.01	*P* < 0.05	NS
L-Tyrosine	203 ± 8	259 ± 9	251 ± 9	463 ± 45	*P* < 0.01	*P* < 0.01	*P* < 0.01
D-Tyrosine	152 ± 12	180 ± 18	285 ± 23	376 ± 10	*P* < 0.01	*P* < 0.01	NS
GABA	60.2 ± 4.2	58.0 ± 2.2	64.9 ± 4.0	75.7 ± 7.5	*P* < 0.05	NS	NS

Values are means ± S.E.M. in pmol/mg wet tissue; *n* = 5–7 in each group; NS: Not significant.

In comparison to the changes in the liver, the number of plasma free amino acids showed less alteration, but all the plasma free amino acids analyzed in the current study are shown in Table 3. L-Serine, taurine, L-alanine, L-valine, D-methionine, L-methionine and L-phenylalanine were significantly (*P* < 0.05) higher in Roborovskii than in Djungarian hamsters, but the reverse was true for L-histidine and D-alanine. L-Serine, L-histidine, L-tryptophan and L-leucine were significantly higher in the night time; however, D-alanine significantly increased in day time. A significant interaction between species and time was detected in L-tryptophan, suggesting that plasma L-tryptophan greatly enhanced during the night time in Roborovskii hamster while there was almost no change in Djungarian hamster.

**Table 3:** **Concentrations of L- and D-amino acids in the plasma of Djungarian and Roborovskii hamsters during light and dark periods**

Amino acids	Djungarian hamster		Roborovskii hamster		Results of ANOVA		
	Light	Dark	Light	Dark	Species	Time	Interaction
*Essential amino acids*							
L-Valine	318 ± 24	316 ± 9	348 ± 37	410 ± 35	*P* < 0.05	NS	NS
L-Histidine	125 ± 4	136 ± 3	105 ± 3	112 ± 9	*P* < 0.01	NS	NS
L-Methionine	75.3 ± 3.7	65.1 ± 4.3	80.6 ± 5.7	87.0 ± 3.6	*P* < 0.01	NS	NS
L-Tryptophan	54.6 ± 3.2	54.0 ± 6.5	25.6 ± 2.7	90.8 ± 7.3	NS	*P* < 0.01	*P* < 0.01
L-Phenylalanine	101 ± 3	104 ± 7	129 ± 2	145 ± 10	*P* < 0.01	NS	NS
L-Leucine	235 ± 17	242 ± 6	206 ± 10	274 ± 25	NS	*P* < 0.05	NS
L-Isoleucine	143 ± 10	142 ± 3	134 ± 10	162 ± 15	NS	NS	NS
*Nonessential amino acids*							
L-Serine	290 ± 27	324 ± 21	374 ± 17	432 ± 13	*P* < 0.01	*P* < 0.05	NS
Taurine	402 ± 49	326 ± 39	467 ± 20	435 ± 45	*P* < 0.05	NS	NS
L-Aspartic acid	19.0 ± 2.5	13.7 ± 0.7	20.6 ± 2.4	21.2 ± 2.6	NS	NS	NS
L-Alanine	579 ± 19	634 ± 57	706 ± 20	847 ± 70	*P* < 0.01	NS	NS
D-Alanine	12.6 ± 2.6	8.0 ± 0.9	7.1 ± 0.8	5.6 ± 0.6	*P* < 0.01	*P* < 0.05	NS
L-Glutamine	956 ± 66	1199 ± 66	1159 ± 18	1176 ± 89	NS	NS	NS
L-Arginine	216 ± 20	247 ± 8	213 ± 14	296 ± 86	NS	NS	NS
D-Methionine	6.23 ± 0.47	6.86 ± 0.69	7.85 ± 0.58	9.71 ± 1.25	*P* < 0.05	NS	NS
L-Tyrosine	102 ± 7	90 ± 7	96 ± 6	105 ± 8	NS	NS	NS
D-Tyrosine	25.9 ± 2.8	20.1 ± 1.5	21.9 ± 0.9	21.7 ± 1.8	NS	NS	NS

Values are means ± S.E.M. in nmol/ml for plasma; *n* = 5–7 in each group; NS: Not significant.

## Discussion

In the present study, we investigated the concentrations of the free L- and D-amino acids between Djungarian and Roborovskii hamsters. Most of the free amino acids were higher in the brain and liver of Djungarian and Roborovskii hamsters at night than day time possibly due to nocturnal food consumption. Moreover, we cannot preclude the possibility of influence of circadian rhythm on the increment of amino acids at night. Circadian rhythms are coordinated by biological clocks (Liu et al. 2007). Li and Lin (2009) reported that circadian metabolic rhythms are fundamental in controlling the nutrient and energy homeostasis. We, therefore, can predict that biological clock dependent circadian rhythm may contribute in the raising of the amino acid concentrations at night in the present study.

ADHD includes hyperactive symptoms that has been studied using animal models of the young spontaneous hypertensive rat (Li et al. 2007), and the DA transporter knockout mouse (Seeman et al. 2007). Some dopaminergic stimulants like amphetamine and methylphenidate are clinically used as the therapeutic drugs for the treatment of ADHD children. Methylphenidate has a therapeutic effect of about 70% in ADHD patients (Cantwell 1996). ADHD model animals have several abnormalities in DA neurotransmission. For example, Roborovskii hamster has low levels of brain DA (Kabuki et al. 2008). It was shown that companion animals such as dogs and cats have also hyperactivity like ADHD symptoms (Vas et al. 2007). In both children and companion animals, however, the use of these drugs raised health problems (Stern and Schell 2012; Weber and Lütschg 2002). Accordingly, it is needed to develop new treatment ways and means to overcome ADHD symptoms.

Amino acid is one of the candidates as a new therapy for hyperactivity. Amino acids, which cannot be completely or fully synthesized by animals, must be supplied in the diet are classified as the essential or indispensable amino acids. However, those that can be synthesized by the animal are termed nonessential or dispensable amino acids (Wu 2009). In addition, some amino acids are important regulators of key metabolic pathways and therefore necessary for several physiological functions (Suenaga et al. 2008; Wu et al. 2007a,[b, c]). They are called functional amino acids, which include tyrosine, serine, arginine, tryptophan, cysteine, glutamine, leucine and proline. Previously, it was reported that the low level of DA in the brain was partly caused the hyperactivity in Roborovskii hamsters (Kabuki et al. 2008), and the administration of L-DOPA and L-tyrosine as DA precursors ameliorated hyperactivities (Kabuki et al. 2009a, 2011). To clarify the reason for low DA levels in the brain of Roborovskii hamster, we first focused on the difference in tyrosine metabolism between Roborovskii and Djungarian hamsters because L-tyrosine is the precursor of DA. In the liver, Roborovskii hamster showed higher levels of both L- and D-tyrosine compared with Djungarian hamster. However, these differences were not reflected in plasma L- or D-tyrosine concentrations. On the other hand, D-tyrosine concentration was significantly higher in the brain of Roborovskii hamsters than Djungarian hamsters. These findings suggest that the increased concentration of D-tyrosine in the brain may not have any correlation with plasma concentration of tyrosine; rather it is regulated by brain or tissue-dependent tyrosine metabolism. To the best of our knowledge, the occurrence of tyrosine racemase has not yet been clarified in any organisms. We are the first to suggest the occurrence of tyrosine racemase in the hamster’s liver and brain. Furthermore, Roborovskii hamster may have a greater activity of tyrosine racemase to convert L-tyrosine to D-tyrosine. Based on our findings, we could predict that if the conversion of L-tyrosine to D-tyrosine was low in Roborovskii hamsters, substrate for DA would be increased and hyperactivity could be moderated. On the other hand, L-phenylalanine, a precursor of L-tyrosine, is higher in the plasma and liver of Roborovskii hamsters; however, it is lower in the brain in this species. This phenomenon also suggests that the L-tyrosine metabolic turnover is higher in the brain of Roborovskii hamsters.

Not only tyrosine metabolism but also serine metabolism was different between Roborovskii and Djungarian hamsters. L-Serine and D-serine in the brain were higher in the Djungarian hamster. D-Serine is known as co-agonist for *N*-methyl-*D*-aspartate (NMDA) receptors (Nishikawa 2005) and stimulation of NMDA receptors induced sedative effects (Yamane et al. 2009). Furthermore, we have reported that L-serine acts as a sedative factor in stress reducing activity in chicks (Asechi et al. 2006, 2008; Shigemi et al. 2008) and rats (Shigemi et al. 2010). Shigemi et al. (2008) revealed the mechanistic process of function of L-serine using the antagonist of γ-aminobutyric acid (GABA)_A_ receptor, picrotoxin where the sedative and hypnotic effects of L-serine was inhibited by the antagonist. Therefore, we can speculate that the presence of high L- and D-serine concentrations in the brain of Djungarian hamster may reduce their activity. Almost all the values of amino acids are nearly the same in the brain and liver among pigs, rats and hamsters. However, some amino acids in the brain were different between hamsters and other mammalian species. L-Tyrosine showed higher levels in the pig (Li et al. 2009), and L-tyrosine, L-serine and L-histidine levels were higher in the rat. Conversely, L-glutamine, GABA, L-valine and L-phenylalanine were lower in rats than in hamsters (Sase et al. 2013).

L-Tryptophan is a precursor of serotonin and serotonin also reduces hyperactivity (Avale et al. 2004). Roborovskii hamsters have lower serotonin level in the brain (Kabuki et al. 2008). In the present study, however, no significant differences were detected in brain L-tryptophan concentration (pmol/mg). This result indicates that metabolic rate of L-tryptophan for the synthesis of serotonin may be slower in Roborovskii hamsters. Wu (2013), Wu et al. (2013b) suggested that as so-called nutritionally nonessential amino acids have profound effects on several important biological functions, they should be included to the diets along with nutritionally essential amino acids to improve food efficiency, growth, and health of animals and humans. Therefore, the nonessential amino acids which were low in the hyperactive Roborovskii hamsters in the current study would be important data for further research to test their effectiveness in hyperactive patients through dietary amino acid supplementations.

In conclusion, metabolism of L-tyrosine to DA pathway may differ between Djungarian and Roborovskii hamsters because D-tyrosine concentration was higher in Roborovskii hamsters. Therefore, Rovorovskii hamsters have a possibility for being considered as a hyperactive model animal due to the dysfunction in tyrosine to DA metabolic pathway. On the other hand, it was clear that Roborovskii hamsters have less L- and D-serine concentrations in the brain, which are known as sedative factors. Taken together, species-specific regulation of amino acid metabolism in the brain may contribute to the hyperactivity in Rovorovskii hamsters.
